# miR-223 functions as a potent tumor suppressor of the Lewis lung carcinoma cell line by targeting insulin-like growth factor-1 receptor and cyclin-dependent kinase 2

**DOI:** 10.3892/ol.2013.1375

**Published:** 2013-06-04

**Authors:** WEIQI NIAN, XUJUN AO, YONGZHONG WU, YI HUANG, JIANGHE SHAO, YIMING WANG, ZHENGTANG CHEN, FANGLIN CHEN, DONGLIN WANG

**Affiliations:** 1 Department of Oncology, Chongqing Tumor Hospital, Chongqing 400030, P.R. China; 2 Institute of Cancer Research, Xinqiao Hospital, Third Military Medical University, Chongqing 400037, P.R. China; 3 Department of General Surgery, 532 Hospital of the People’s Liberation Army, Huangshan, Anhui 245041, P.R. China; 4 Center for Clinical Molecular Medicine, Children’s Hospital, Chongqing Medical University, Chongqing 400014, P.R. China

**Keywords:** non-small cell lung cancer, miR-223, tumor suppressor, metastasis, self-renewal

## Abstract

microRNAs (miRNAs) have been hypothesized to function as oncogenes or tumor suppressors by targeting specific cancer-related genes. Previous studies have reported that miR-223 may serve as a tumor suppressor in a number of cancer types, however, knowledge of its targets in non-small cell lung cancer (NSCLC) remains limited. In the current study, miR-223 was found to inhibit cell proliferation *in vitro* by CCK-8 assay, growth curves and an anchorage-independent growth assay in a Lewis lung carcinoma (LLC) cell line. miR-223 transfection in the LLC cells was observed to significantly inhibit migration and invasion, induce G_2_/M arrest and decrease the expression levels of Sca-1, a marker of murine stem cells. In addition, miR-223 transfection markedly suppressed AKT and ERK signaling, as well as insulin-like growth factor-1 receptor (IGF-1R)-mediated downstream signaling, pathways that are crucial for cell proliferation and invasion in NSCLC cells. Analyses in C57BL/6 mice demonstrated that miR-223 suppresses tumorigenicity *in vivo*. Using a luciferase activity assay and western blot analysis, IGF-1R and cyclin-dependent kinase 2 (CDK2) were identified as direct targets of miR-223. In the present study, novel cancer-related targets of miR-223 were identified and verified in a LLC cell line, indicating that miR-223 functions as a tumor suppressor, which may fine-tune the activity of the IGF-1R pathway in lung cancer. Therefore, increasing miR-223 expression may provide a novel approach for the treatment of NSCLC.

## Introduction

Lung cancer is the most common cancer worldwide in terms of incidence and mortality, and its incidence is rapidly increasing in developing countries (1–2). The prognosis of lung cancer remains poor despite recent advances in chemotherapies and molecular-targeted therapies. The five-year survival rate of lung cancer is <15%, and ∼90% of mortalities are caused by metastasis. To improve patient survival, the elucidation of the regulatory mechanisms that control the tumor metastatic properties of lung cancer is urgently required.

microRNAs (miRNAs) are small non-coding RNA molecules that suppress gene expression by interacting with the 3′ untranslated regions (UTRs) of target mRNAs (3). Although miRNAs account for only a minor fraction of the expressed genome, these RNAs are involved in modulating a number of cellular pathways, including proliferation (4), differentiation (5) and apoptosis (6). The deletion or epigenetic silencing of miRNAs that normally repress the expression of one or more oncogenes may lead to carcinogenesis (7). Thus, miRNAs have been hypothesized to function as tumor suppressors or oncogenes, and alterations in miRNA expression may be critical for tumorigenesis and cancer progression (8–10). miR-223 is a highly conserved miRNA and is crucial for triggering the myeloid differentiation of progenitor cells (11) and for maintaining granulocyte function (12). Previous studies have reported a number of significant miR-223 targets associated with malignancy, including insulin-like growth factor-1 receptor (IGF-1R) (13), myocyte enhancer factor 2C (14), stathmin 1 (15), artemin (16) and FOXO1A (17). miR-223 has been reported to be markedly downregulated in the lungs of rats exposed to environmental cigarette smoke for 28 days (18). The reduced serum expression of miR-223 was found to be associated with cancer-specific mortality in stage IA/B patients (19). In our previous study, CXCR4-positive cells from the Lewis lung carcinoma (LLC) cell line presented with cancer metastatic stem cell characteristics, and miR-223 expression was reduced compared with in CXCR4-negative cells (20,21). However, the mechanism by which miR-223 functions in the development of NSCLC remains largely unknown and, to date, no miR-223 targets have been reported in NSCLC. Therefore, in the present study, the effects of miR-223 transfection in the LLC cell line were evaluated to determine whether miR-223 functions as a tumor suppressor of lung cancer.

## Materials and methods

### Cell line and culture

The LLC cell line was purchased from the American Type Culture Collection (Manassas, VA, USA). The LLC cells were cultured in Dulbecco’s modified Eagle’s medium (DMEM) with 10% fetal calf serum, 100 U/ml streptomycin and 100 U/ml penicillin in a humidified atmosphere of 95% air and 5% CO_2_ at 37°C.

### Cell transfection

For the functional analysis, miR-223 and non-targeting miRNA mimics (Thermo Fisher Scientific, Inc., Waltham, MA, USA) were used. For the target validation and cell signaling analysis, the mmu-miRNA-223 expression vector and an empty pGenesil-1.1 (Wuhan Genesil Biotechnology Co Ltd., Hubei, China) were used. The mimics were transfected into the appropriate cells using Lipofectamine 2000 (Invitrogen Life Technologies, Carlsbad, CA, USA) according to the manufacturer’s instructions.

### Cell survival assays

The effects of miRNA-223 expression on LLC proliferation were assessed using Cell Counting Kit-8 (CCK-8; Dojindo Laboratories, Kumamoto, Japan). Briefly, the cells were plated on 96-well plates. Following transfection, CCK-8 was added to each well at various times and incubated at 37°C for 1.5 h. The absorbance at 450 nM was measured using a microplate spectrophotometer (Tecan Group Ltd, Männedorf, Switzerland).

### Anchorage-independent growth ability assay

In total, 500 cells were trypsinized and suspended in 2 ml complete medium with 0.3% agar (Sigma-Aldrich, St Louis, MO, USA). The agar-cell mixture was plated on top of a bottom layer with 1% complete medium agar mixture. Subsequent to 10 days, the viable colonies containing >50 cells or those >0.1 mm in size were counted. The colony size was measured using an ocular micrometer.

### Invasion assay

The LLC measurements were performed in 24-well matrigel-coated invasion chambers. The lower chambers contained 600 *μ*l DMEM with 10% fetal bovine serum (FBS) as a chemoattractant. At 24 h post-transfection with miRNA-223 or non-targeting miRNA mimics, a cell suspension of 5×10^4^ cells in 100 *μ*l DMEM with 0.5% FBS was added to the upper chamber. Following incubation for 24 h at 37°C in a humidified incubator with 5% CO_2_, the invasive cells that had attached to the lower surface of the membrane insert were fixed by 4% formaldehyde and stained with crystal violet. The number of cells was then quantified under a microscope.

### Wound healing assay

For the wound healing assay, the LLC cells were grown to confluence on 6-well plates. Next, linear scratch wounds were created on the confluent monolayer using a pipette tip and the cells were transfected with 50 nM miR-223 mimics or 50 nM non-targeting miRNA mimics (control). Floating cells were removed by gentle washes with culture medium. The healing process was examined dynamically and was recorded with a digital camera 24 h after wound generation.

### Matrix metallopeptidase (MMP)9 enzyme-linked immunosorbent assay (ELISA)

Following transfection with miRNA-223 or non-targeting miRNA mimics, MMP9 protein levels in the culture supernatants were quantified using an ELISA kit according to the manufacturer’s instructions (R&D Systems, Minneapolis, MN, USA). Plates were read at 450 nM using a Synergy HT microplate reader (BioTek Instruments, Inc., Winooski, VT, USA) and the MMP9 concentration in the samples was calculated using a standard curve.

### Flow cytometric analysis

At 48 h post-transfection with miRNA-223 or non-targeting miRNA mimics, dissociated LLC cells were stained with PE-conjugated (PE) rat anti-mouse Sca-1 and the corresponding isotype controls (1:100; BD Pharmingen, San Diago, CA, USA). Briefly, the cells were trypsinized; the trypsin was neutralized with culture medium containing adult bovine serum (ABS) and centrifuged at 450 × g for 5 min. The cell pellet was re-suspended in Hank’s buffered salt solution (HBSS) containing 2% ABS (HBSS^+^). The cells were incubated with primary antibodies for 20 min on ice, washed twice with HBSS^+^ and re-suspended with HBSS^+^ containing Sytox Blue or Sytox Red (Invitrogen Life Technologies) to exclude any dead cells. The cell suspensions were filtered using 40-*μ*m filters and analyzed on a BD LSRII Fortessa (both BD Biosciences, Franklin Lakes, NJ, USA). In addition, a cell cycle analysis was performed as described previously (22).

### In vivo tumorigenicity

Female C57BL/6 mice (6–8 weeks old) were obtained from The Animal Facility of The Third Military Medical University (Chongqing, China). *In vivo* experiments were performed in accordance with institutional guidelines. This study was approved by the ethics committee of Chongqing Tumor Hospital, Chongqing, China. Two groups of mice were tested. Group 1 (223-mimic) was injected with LLC cells transfected with miR-223 mimics and group 2 (mimic-control) was injected with LLC cells transfected with non-targeting miRNA mimics. The mice were sacrificed at four weeks, and the tumor volume was calculated using the following formula: [L × (W)^2^] / 2, where L is the length and W is the width of the tumor. For the immunofluorescence labeling, the cells were blocked with 15% bovine serum albumin (BSA) for 1 h and then incubated at 37°C for 1 h in PE-conjugated rat anti-mouse Sca-1 (1:100). Following this, the cells were washed with 0.01% PBS and then stained with 4,6-diamidino-2-phenylindole (Sigma-Aldrich) to identify the cell nuclei. The cells were then observed under a fluorescence microscope.

### Luciferase reporter plasmid construction and luciferase assay

The pMIR-REPORT miRNA expression reporter (firefly luciferase reporter plasmid; Life Technologies, Grand Island, NY, USA) was used for the plasmid construction. Constructs were generated using the following primers: IGF-1R 3′-UTR-1, 5′-GGACTAGTAGGGGAGAGCAGGTTG TAACAATCT-3′ and 5′-CGACGCGTGACCTACGGTGTC AGGCAGGTGTAT-3′; IGF-1R 3′-UTR-2, 5′-GGACTAGTC AGTACCTGACAGTAGGCCAATGAT-3′ and 5′-CGACGC GTAAGATTTGGTCAGTCCTTGTTTAGC-3′; cyclin-dependent kinase (CDK)2 3′-UTR: 5′-GGACTAGTAGCCTTCTGA TGTTTTCTGGCTGTC-3′ and 5′-CGACGCGTGATGAAC AGACCAGAGTGACGTGCA-3′. The 3′-UTR and miR-223 complementary sequence (TGGGGTATTTGACAAACT GACA) were separately cloned into the pMIR-REPORT plasmid (Life Technologies), according to the manufacturer’s instructions. Constructs (0.05 *μ*g each) were cotransfected into 293T with 0.01 *μ*g a Renilla luciferase control vector using calcium phosphate transfection. Luciferase activity was measured 36 h after transfection and normalized against Renilla activity, according to the manufacturer’s instructions (Dual-Luciferase Reporter Assay System; Promega Corporation, Madison, WI, USA).

### Quantitative (q)PCR

To determine the gene expression levels, qPCR was performed using the Quantitect SYBR PCR kit (Qiagen, Hilden, Germany), according to the manufacturer’s instructions. The primers selected were as follows: IGF-1R forward, 5′-AAGCCGATGTGTGAGAAGACC-3′ and reverse, 5′-ATAGTAGTAGTAGTG GCGGCAAGC-3′; CDK2 forward, 5′-TTCATGGATGCCTCTGCTCTC-3′ and reverse, 5′-TCC AAAAGCTCTGGCTAGTCC-3′; MMP9 forward, 5′-GTG GAGAGTCGAAATCTCTGG-3′ and reverse, 5′-TTTGGA ATCTGCCCAGGTCTG-3′; GAPDH forward, 5′-TGGTAT CGTGGAAGGACTCATGAC-3′ and reverse, 5′-ATGCCA GTGAGCTTCCCGTTCAGC-3′. ΔΔC_T_ values were normalized against those obtained from the amplification of GAPDH. All reactions were performed in triplicate.

### Western blot analysis

Transfected cells in culture were harvested at various times, washed once with cold PBS and lysed in buffer containing protease inhibitors. The protein concentrations from whole cultured cells were measured with the bicinchoninic acid protein assay kit (Beyotime Institute of Biotechnology, Shanghai, China), using BSA as the standard. Protein (30 *μ*g) was separated by SDS-PAGE using a 10% polyacrylamide gel and then electroblotted onto a nitrocellulose membrane. The membrane was immunoblotted overnight at 4°C with the primary antibodies. The following antibodies were used at a 1:1,000 dilution: anti-IGF-IR, anti-mmp9 (Santa Cruz Technologies, Santa Cruz, CA, USA), anti-phospho-IGFIR, anti-phospho-Akt, anti-Akt, anti-p44/42 MAPK (Erk1/2), anti-phospho-Erk1/2 and anti-CDK2 (Cell Signaling Technology, Beverly, MA, USA). A goat anti-rabbit horseradish peroxiadse (HRP) secondary antibody (Wuhan Boster Bio-Engineering Co, Ltd, Wuhan, China) was used at a 1:2,000 dilution. The proteins were detected using an enhanced chemiluminescence kit (Pierce Biotechnology, Inc., Rockford, IL, USA), according to the manufacturer’s instructions. GAPDH was used as an internal control.

### Statistical analysis

Data are presented as the mean ± SD and analyzed using SPSS 16.0 (SPSS, Inc., Chicago, IL, USA). To assess the statistical significance of the differences, an unpaired t-test was performed. P<0.05 was considered to indicate a statistically significant difference.

## Results

### Upregulation of miRNA-223 suppresses proliferation and tumorigenicity of LLC cells

**Figure 1.**
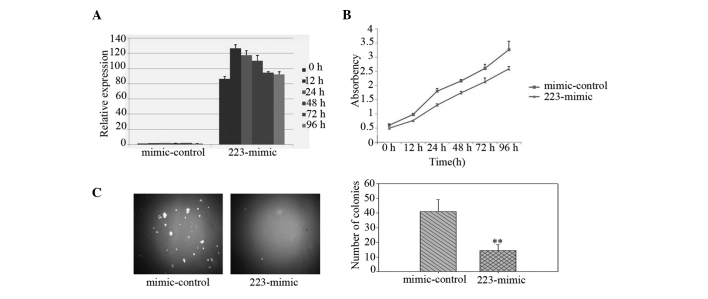
miR-223 overexpression reduces proliferation and clonogenic cell growth. (A) Effect of miR-223-mimic transfection into LLC cells was confirmed by quantitative (q)PCR. (B) Growth curves of miR-223- and control mimic-transfected LLC cells were conducted by CCK-8 assay. (C) Upregulation of miR-223 inhibited LLC tumorigenicity as determined by anchorage-independent growth assay. Magnification, ×100. Data are presented as the mean ± SD of three replicates. ^**^P<0.01 vs. mimic-control. miR, microRNA; LLC, Lewis lung carcinoma; CCK-8, Cell Counting kit-8.

To investigate the biological role of miR-223 expression in the development and progression of lung cancer, LLC cells were transfected with miR-223 mimics and the effect on cellular proliferation was assessed. Following transfection, the miR-223 levels were increased in the LLC cells, indicating that the increase was due to miR-223 transfection (Fig. 1A). Using a CCK-8 assay, the overexpression of miR-223 (223-mimic) was observed to markedly reduce the growth rate of the LLC cells compared with that of the non-targeting miRNA mimic-transfected cells (mimic-control; Fig. 1B). Notably, the LLC cells ectopically expressing miR-223 were identified to exhibit a significantly inhibited anchorage-independent growth ability, as demonstrated by the decrease in colony numbers and sizes (Fig. 1C), indicating that upregulation of miR-223 reduces the tumorigenicity of lung cancer cells *in vitro*.

### Ectopic expression of miRNA-223 inhibits LLC invasion

**Figure 2.**
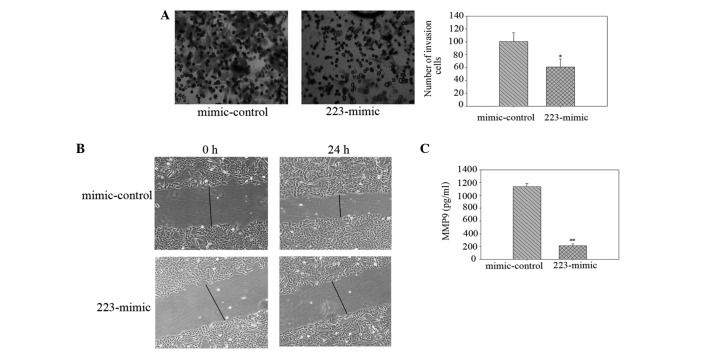
miR-223 overexpression inhibits invasion in LLC cells. (A) Invasion assay for miR-223- and control mimic-transfected cells. Magnifcation, ×200. Relative quantification of invaded cells from transwell invasion assay are presented. (B) Representative images of wound healing (24 h after scratch) in miR-223 and control mimic-transfected cells are presented. Magnifcation, ×200. (C) At 48 h post-transfection, expression of MMP-9 protein in culture supernatants was estimated by enzyme-linked immunosorbent assay (ELISA). ^*^P<0.05 and ^**^P<0.01, vs. mimic-control. miR, microRNA; LLC, Lewis lung carcinoma.

To examine invasion, the LLC cells were transfected with miR-223 or control mimics and reseeded on top of the insert. Subsequent to 48 h, the number of transmembrane cells in the 223-mimic group (60.67±12.66) was lower than that of the mimic-control group (100.33±14.01; P<0.05; Fig. 2A). Next, the LLC cells were transfected as described, scratch wounds were generated and cell migration towards the wound was visualised. The wound healing assay revealed that miR-223 reduced the motility of the LLC cells (Fig. 2B). To determine whether the increased invasion observed was correlated with concomitant changes in MMP levels, the total active MMP9 protein levels were measured by ELISA in the cultured media. The MMP9 levels in the supernatant of miR-223-overexpressing cells (214.16±28.18 pg/ml) were reduced compared with that of the mimic-controls (1,139.14±50.13 pg/ml; P<0.01; Fig. 2C). These observations indicated that miR-223 inhibits invasion in LLC cells.

### Overexpression of miR-223 in LLC cells induces G_2_/M phase arrest and reduces Sca-1 protein expression

**Figure 3.**
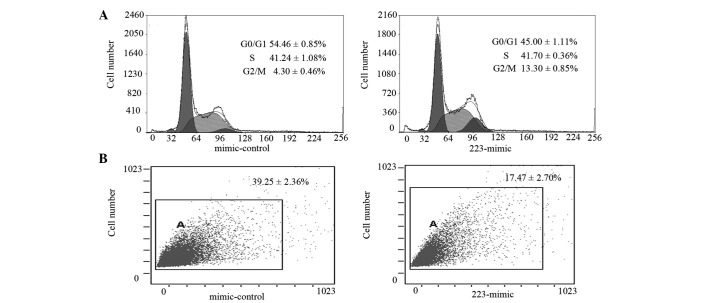
miR-223 affects the cell cycle and self renewal in LLC cells. (A) Fluorescence-activated cell sorting (FACS) analysis of the cell cycle status of 223-mimic and mimic-control transfected cells. (B) FACS analysis of the Sca-1 expression in 223-mimic and mimic-control transfected cells. Data are presented as the mean ± SD of three replicates. miR, microRNA; LLC, Lewis lung carcinoma.

Propidium iodide staining of miR-223-overexpressing LLC cells revealed an increase in the G_2_/M cell populations (13.3±0.85 vs. .30±0.46%) and a decrease in cells in the G_0_/G_1_ phase populations (45.00±1.11 vs. 54.46±0.85%) compared with the mimic-control (P<0.01; Fig. 3A), indicating a block in the G_2_/M phase transition of the cell cycle. Notably, the percentage of Sca-1-positive cells (Sca-1 is a well-known marker in murine stem cells) was reduced from 39.25±2.36 to 17.47±2.70% in the miR-223-overexpressing group (P<0.01; Fig. 3B).

### miR-223 expression in LLC inhibits tumor growth in vivo

**Figure 4.**
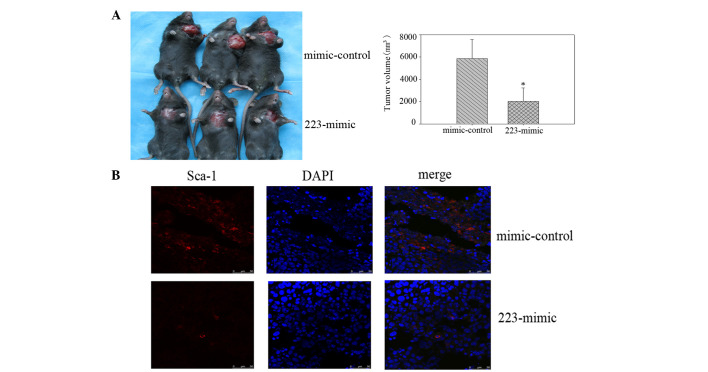
*In vivo* functional studies on the effect of miR-223-treatment on LLC cells in C57BL/6 mice. (A) Decreasing trend in size of tumors from miR-223- to control mimic-transfected cells. ^*^P<0.05 vs. mimic-control. (B) Sca-1 expression was analyzed by fluorescence microscopy of LLC tissues treated with miR-223 and control mimics. Cell nuclei were stained with 4,6-diamidino-2-phenylindole (magnifcation, ×100). miR, microRNA; LLC, Lewis lung carcinoma.

At four weeks post-injection, the mice administered with miR-223 mimics had formed markedly smaller tumors than the mimic-control group (Fig. 4A). The tumor volume subsequent to sacrifice in mice injected with miR-223 mimic-transfected cells was 2,034.30±983.99 mm^3^, whereas the tumor volume in mice injected with the control mimic-transfected cells was 5,860.20±692.58 mm^3^. The Sca-1 expression in the tumor tissue from the miR-223 mimic-transfected cells was significantly lower than that of the control mimic-transfected cells, as demonstrated by immunofluorescence staining (Fig. 4B).

### miR-223 regulates IGF-1R and CDK2 levels by binding to the 3′UTR

**Figure 5.**
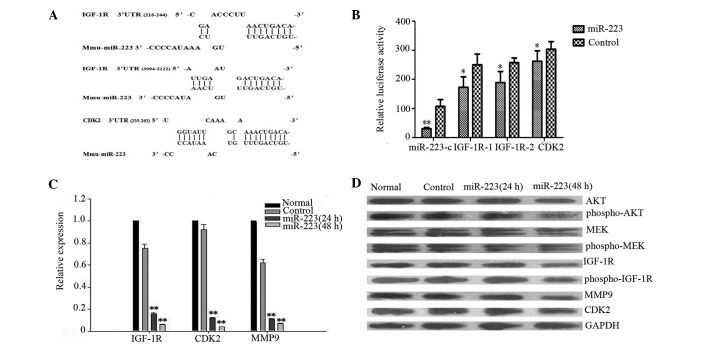
miR-223 regulates the activity of the IGF-1R signaling pathway and the expression of its key genes in LLC cells. (A) Sequence alignment of the miR-223 seed region and mRNA targets. (B) Luciferase 3′-UTR reporter assays of miR-223-induced gene silencing effects. (C) Expression of IGF-1R, CDK2 and MMP9 mRNA was significantly suppressed by transfection of cells with pre-miR-223, as confirmed by quantitative (q)PCR in LLC. (D) Detection and comparison of IGF-1R, CDK2, MMP9 and multiple phosphorylated kinase expression was altered following transfection with miR-223 or control vector by western blot analysis. GAPDH was used as loading control. ^*^P<0.05, ^**^P<0.01 vs. control. All data are representative of three independent experiments. miR, microRNA; LLC, Lewis lung carcinoma; UTR, untranslated region.

Bioinformatic analyses revealed that the IGF-1R 3′UTR contained two putative miR-223 binding sites for miR-223 and CDK2 contained one putative miR-223 binding site for miR-223 (Fig. 5A). The luciferase activities of the IGF-1R 3′UTR, CDK2 3′UTR and miR-223 complementary sequence-containing constructs were found to be further repressed in cells overexpressing miR-223 (Fig. 5B). Next, to examine whether miR-223 affects IGF-1R and CDK2 expression in LLC, the mRNA and protein expression levels of IGF-1R and CDK2 were analyzed using qPCR and western blot analysis. Subsequent to 48 h, the expression of IGF-1R and CDK2 mRNA in the miR-223-expressing group was reduced by ∼17- and 25-fold of the control vector group, respectively (Fig. 5C). miR-223 also caused a significant reduction in the IGF-1R and CDK2 protein levels (Fig. 5D). These results indicate that IGF-1R and CDK-2 are post-transcriptionally regulated by miR-223 in LLC cells.

Two pathways have been described for IGF-IR, the phosphatidylinositol-3 kinase-Akt and mitogen-activated protein kinase pathways (also known as ERKs) (23). To determine the consequences of the interference of IGF-1R expression by miR-223, the expression of IGF-1R, Akt, ERK and their active forms (p-IGF-1R, p-Akt and p-ERK) was measured. The expression of IGF-1R, p-IGF-1R, p-Akt and p-ERK was reduced, however, the total Akt and ERK levels were unaffected (Fig. 5D). The downregulation of MMP9 expression at the mRNA and protein levels was further supported by qPCR and western blot analysis (Fig. 5C and D). These results indicate that the IGF-1R-mediated downstream signaling pathway was also affected by miR-223.

## Discussion

The current study is the first study to demonstrate that miR-223 may be involved in lung cancer stem cell self-renewal and to show that miR-223 functions as a tumor suppressor in lung cancer cells at multiple steps of tumorigenesis and progression. In addition, IGF-1R and CDK2 were demonstrated to represent two significant targets for miR-223, crucial for mediating the miR-223-regulated malignant phenotype of LLC cells.

In the present study, IGF-1R expression and its phosphorylation levels in LLC were investigated and found to be markedly reduced, while cell proliferation was inhibited, following miR-223 overexpression. In addition, analyses using a IGF-1R 3′UTR reporter revealed a significant decrease in luciferase activity. In conclusion, these observations demonstrate that IGF-1R is the functional target of miR-223. IGF-1R is a transmembrane receptor tyrosine kinase encoded by a gene located on chromosome 15q26.3. IGF-1R is implicated in the promotion of oncogenic transformation, growth and the survival of cancer cells (24,25). It has been previously reported that IGF signaling mediates the transformation of normal lung cells and is involved in tumor initiation (26,27). In addition, a number of studies in human lung cancer cell lines have demonstrated that the downregulation of IGF-IR inhibits lung tumor cell proliferation and sensitizes lung cancer cells to chemotherapy and radiotherapy (28,29).

miR-223 has also been found to also target the CDK2 3′UTR. CDK2 is an S-phase cyclin-dependent kinase that is required for p53-independent G_2_/M checkpoint control. The inhibition of cyclin A/cdk2 activation contributes to the maintenance of G_2_ phase arrest in response to DNA damage (30,31). In human leukemia cells, inhibitors of ERK have been reported to increase the phosphorylation of cdc25c expression at the G_2_/M arrest stages and decrease p21 and CDK2 expression at the endoreduplication stages (22). Previous studies have demonstrated that the cyclolignan, picropodophyllin, downregulates IGF-1R tyrosine kinase activity and induces a marked accumulation of cells in the G_2_/M-phase, as well as increased apoptosis (32). These observations, together with the results of the present study, are consistent with a model in which miR-223 induces G_2_/M arrest via the downregulation of IGF-1R and CDK2 by co-targeting their 3′UTR regions.

The present study primarily focused on whether the IGF-1R-mediated downstream signaling pathway is affected by miR-223. The ERK and PI3K/Akt signaling pathways are central to the regulation of MMP9 expression (33,34). The present results indicated that miR-223-decreased MMP9 activity was mediated by the suppression of phospho-ERK1/2 or phospho-Akt. In addition, the forced expression of miR-223 was found to downregulate Sca-1 in the LLC cells, as well as a simultaneous decrease of the cells in the G_0_/G_1_ phase and the induction of the inhibition of anchorage-independent growth. Sca-1, or Ly6A, is a member of the Ly6 family of glycosyl phostidylinositol-anchored cell surface proteins that is associated with murine stem/progenitor cells (35,36). The MAPK/ERK pathway is essential for Sca-1^+^ hepatic progenitor cell proliferation and colony formation (37). In addition, IGF-1 stimulation directly induces anoikis resistance of a number of varying epithelial cell types by activating downstream signaling molecules, including Ras/MAPK and PI3K/Akt (38). In a colon cancer model, the kinase activities of Akt and ERK1/2 were shown to be significantly upregulated in CD133^+^ cells (39). The clonogenic growth of the CD133^+^ cells was reduced markedly by inhibiting the activity of AKT and ERK1/2. In agreement with these results, we hypothesize that miR-223 induces an aberrant self-renewal capacity in LLC cells, at least in part, via the inhibition of AKT and ERK activity. Future studies must be performed to verify this hypothesis and identify the underlying mechanisms.

In the current study, miR-223 was revealed to function as a tumor suppressor in lung cancer. The results also indicate that miR-223 may fine-tune the activity of the IGF-1R pathway. These observations may provide a basis for novel therapies targeting IGF-1R in the treatment of NSCLC.
